# Standard‐space atlas of the viscoelastic properties of the human brain

**DOI:** 10.1002/hbm.25192

**Published:** 2020-09-15

**Authors:** Lucy V. Hiscox, Matthew D. J. McGarry, Hillary Schwarb, Elijah E. W. Van Houten, Ryan T. Pohlig, Neil Roberts, Graham R. Huesmann, Agnieszka Z. Burzynska, Bradley P. Sutton, Charles H. Hillman, Arthur F. Kramer, Neal J. Cohen, Aron K. Barbey, Keith D. Paulsen, Curtis L. Johnson

**Affiliations:** ^1^ Department of Biomedical Engineering University of Delaware Newark Delaware USA; ^2^ Thayer School of Engineering Dartmouth College Hanover New Hampshire USA; ^3^ Beckman Institute for Advanced Science and Technology University of Illinois at Urbana‐Champaign Urbana Illinois USA; ^4^ Interdisciplinary Health Sciences Institute University of Illinois at Urbana‐Champaign Urbana Illinois USA; ^5^ Département de génie mécanique Université de Sherbrooke Sherbrooke Québec Canada; ^6^ College of Health Sciences University of Delaware Newark Delaware USA; ^7^ School of Clinical Sciences University of Edinburgh Edinburgh UK; ^8^ Carle Neuroscience Institute Carle Foundation Hospital Urbana Illinois USA; ^9^ Department of Human Development and Family Studies and Molecular, Cellular and Integrative Neurosciences Colorado State University Fort Collins Colorado USA; ^10^ Department of Bioengineering University of Illinois at Urbana‐Champaign Urbana Illinois USA; ^11^ Department of Psychology Northeastern University Boston Massachusetts USA; ^12^ Department of Physical Therapy, Movement, & Rehabilitation Sciences Northeastern University Boston Massachusetts USA

**Keywords:** brain atlases, magnetic resonance elastography, magnetic resonance imaging, mechanical properties, MRI templates, viscoelasticity

## Abstract

Standard anatomical atlases are common in neuroimaging because they facilitate data analyses and comparisons across subjects and studies. The purpose of this study was to develop a standardized human brain atlas based on the physical mechanical properties (i.e., tissue viscoelasticity) of brain tissue using magnetic resonance elastography (MRE). MRE is a phase contrast‐based MRI method that quantifies tissue viscoelasticity noninvasively and in vivo thus providing a macroscopic representation of the microstructural constituents of soft biological tissue. The development of standardized brain MRE atlases are therefore beneficial for comparing neural tissue integrity across populations. Data from a large number of healthy, young adults from multiple studies collected using common MRE acquisition and analysis protocols were assembled (N = 134; 78F/ 56 M; 18–35 years). Nonlinear image registration methods were applied to normalize viscoelastic property maps (shear stiffness, *μ*, and damping ratio, *ξ*) to the MNI152 standard structural template within the spatial coordinates of the ICBM‐152. We find that average MRE brain templates contain emerging and symmetrized anatomical detail. Leveraging the substantial amount of data assembled, we illustrate that subcortical gray matter structures, white matter tracts, and regions of the cerebral cortex exhibit differing mechanical characteristics. Moreover, we report sex differences in viscoelasticity for specific neuroanatomical structures, which has implications for understanding patterns of individual differences in health and disease. These atlases provide reference values for clinical investigations as well as novel biophysical signatures of neuroanatomy. The templates are made openly available (github.com/mechneurolab/mre134) to foster collaboration across research institutions and to support robust cross‐center comparisons.

## Graphical abstract

Tissue mechanical properties provide a macroscopic representation of the microstructural constituents of soft biological tissue. In the work, we have produced the first standard‐space atlas description of the stiffness and damping ratio of the healthy human brain. The detailed nature of the new atlas has revealed that neuroanatomical structures possess distinct mechanical characteristics and that sex differences exist in a range of brain structures. Our results provide a novel biophysical signature of the brain and have implications for understanding individual differences in both health and disease.

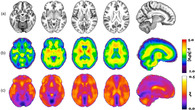

## 1 INTRODUCTION

Brain atlases for structural magnetic resonance imaging (MRI) are important tools for neuroimaging research. Individual scans from multiple participants can be combined to form an anatomical representation of the brain that may reveal group‐wise or study population features. To enable these analyses, a spatial normalization process, or brain registration, is required to transform images into a standardized, or “stereotaxic,” 3D coordinate frame. Once data from each participant is transformed to a common space, anatomical MRI atlases may also be used as registration targets for segmentation, determining functional activation, and statistical mapping, and should be defined for specific age groups (Dickie et al., 2017). Standardized atlases have also been created based on more advanced forms of quantitative imaging such as diffusion weighted imaging (DWI; Mori et al., 2008; Peng et al., 2009; Zhang, Wu, et al., 2018), which, through the assessment of water diffusion rate (Basser, Mattiello, & LeBihan, 1994), can quantify the properties of the white matter microstructure as well as reconstruct neuroanatomical fiber tracts. For example, Zhang, Wu, et al. (2018) created a tract‐based white matter atlas from 100 participants and annotated a total of 256 white matter structures to enable white matter tract parcellations across different populations. Corresponding atlases can also be produced for other quantitative MRI techniques such as magnetization transfer, spectroscopy, contrast enhanced MRI, blood perfusion, and myelin water imaging, for which exact physical or chemical variables can be extracted (Pierpaoli, 2010).

Currently lacking in the literature, however, is a comprehensive, standardized atlas of the brain's mechanical properties (i.e., viscoelasticity). A detailed characterization of physical parameters such as shear stiffness and damping ratio (the viscous behavior of brain tissue) will provide important information for understanding the composition and organization of cells and the extracellular matrix. Magnetic resonance elastography (MRE; Muthupillai et al., 1995) is a relatively new technique that can noninvasively measure these mechanical parameters, expressed in terms of the complex‐valued shear modulus, G*, which is highly sensitive to microstructural tissue integrity (Sack, Johrens, Wurfel, & Braun, 2013). MRE has already been applied in a wide range of neurodegenerative and neurological disorders that have illustrated the sensitivity of MRE for characterizing mechanical alterations due to expected neuropathology (Hiscox et al., 2016; Johnson & Telzer, 2018; Murphy, Huston 3rd, & Ehman, 2019). Few studies, however, have sought to provide comprehensive, standard‐space maps and values for mechanical properties in healthy young adults as the sample sizes in brain MRE reports have previously been limited. Prior publications in brain MRE often emphasize technical advances in the method at the expense of sample size, with a typical sample including between 10 and 30 participants, save for a few exceptions. An early effort provided some of the first standard‐space images; however, the sample size was limited (*N* = 23), MRE data were collected from participants over a wide age range, and properties from few regions were reported (Guo et al., 2013). The examination of possible sex differences in brain viscoelasticity is also under‐explored with brain MRE, which may have implications for understanding patterns of individual differences in both health and disease. In previous work, Sack et al. (2009) reported that female brains are 9% less viscous than males across the lifespan, whereas Arani et al. (2015) reported that the temporal and occipital lobes are stiffer in females than males in older age. A more detailed understanding of differences in brain viscoelasticity between sexes, through the assessment of specific neuroanatomical structures and utilizing a substantially larger sample size, may inform the appropriate design and statistical analyses in future MRE investigations.

Motivated by the growing interest in brain MRE and the vast amount of information that is rapidly accumulating about brain tissue biomechanics, the primary object of this study was to create a publicly available, representative, in vivo template of the mechanical properties of the healthy human brain in a young adult population. To do so, we assembled T1‐weighted structural images and high‐resolution MRE data from 134 participants from multiple sites and studies based on common imaging and inversion protocols. To take full advantage of the abundant information available from these atlases, our secondary object was to complete a comprehensive analysis of the mechanical properties of various brain structures. We are particularly interested in how specific structures within subcortical gray matter, white matter tracts, and parcellations of the cerebral cortex may differ in their viscoelasticity thereby revealing a novel biophysical signature of anatomy. Furthermore, consideration of sex as a biological variable may offer additional insight into individual differences in brain tissue microstructure that may relate to functional or behavioral outcomes. We suggest that the average properties and inherent population variability provided will improve the diagnostic value of brain MRE and may also be used to enhance biomechanical modeling and computer simulations of the brain's response to impact underlying traumatic brain injury and for computer‐integrated neurosurgical systems.

## 2 MATERIALS AND METHODS

### 2.1 Participants

MRI and MRE data from a total of 134 healthy young participants aged between 18 and 35 years (78 female, 23.0 ± 4.4; 18–35 years; 56 male, 24.6 ± 4.3, 18–33 years) were assembled from studies conducted at the University of Edinburgh, UK (UoE), University of Illinois at Urbana‐Champaign; IL, USA (UIUC), and the Carle Foundation Hospital; Urbana, IL (CFH). Criteria for exclusion included history or current diagnosis of a severe medical, neurological, or psychiatric disorder, history of major head injury, and contraindications for undergoing MRI (such as claustrophobia or the presence of an implanted pacemaker). All participants had provided written informed consent according to procedures approved by the institutional committee for the protection of human participants at the respective institutions.

### 2.2 Imaging acquisition

All scans were performed on a Siemens 3T MRI scanner, including both Trio and Verio models (Siemens Healthineers; Erlangen, Germany). Each imaging session comprised an MRE acquisition and a high‐resolution, T1‐weighted 3D magnetization‐prepared rapid acquisition gradient echo (MPRAGE) acquisition that was used in the normalization procedure (see following section). Each MRE acquisition employed a 3D multislab, multishot spiral sequence to capture high‐resolution displacement data (Johnson et al., 2014) and an auxiliary scan for estimating magnetic field inhomogeneity (Funai, Fessler, Yeo, Olafsson, & Noll, 2008). In the majority of studies, MRE data were acquired at an isotropic resolution of 1.6 mm; in a small minority of participants, MRE data were acquired at a 2.0 mm isotropic resolution (*N* = 31). For all studies, a commercially available actuator system (Resoundant; Rochester, MN) was used to elicit brain tissue displacements for MRE at a single frequency of 50 Hz. Vibrations were generated by the active driver situated in the MRI equipment room and transferred through a pneumatic hose to a soft pad placed below the head. The resulting tissue deformation from the applied motion was encoded using motion‐sensitizing gradients (MEGs) embedded in the MRE sequence, which was repeated to capture motion along three orthogonal axes with opposite gradient polarities and through four phase offsets to observe wave propagation in time. Relevant imaging parameters for the MPRAGE and MRE sequences used across different studies are presented in Table 1.

**TABLE 1:** Summary of subject demographics and imaging parameters used across studies

Study	A	B	C	D	E
*N*	12	19	6	31	66
Site	UoE	UIUC	CFH	UIUC	UIUC
Scanner model	Verio	Trio	Trio	Trio	Trio
No. of coils	12	32	12	12	32
*MPRAGE*					
*TE* (ms)	2.97	2.32	2.32	2.32	2.32
*TR* (ms)	2,400	1900	1900	1900	1900
Resolution (mm^3^)	1.0	0.9	0.9	0.9	0.9
*MRE*					
Frequency (Hz)	50	50	50	50	50
Sequence	Spiral	Spiral	Spiral	Spiral	Spiral
Resolution (mm^3^)	1.6	1.6	1.6	2.0	1.6
No. of slices	60	60	60	60	60

*Note:* Data from these studies have previously been published elsewhere, see Study A = Hiscox et al., 2018; Hiscox, Johnson, McGarry, Marshall, et al., 2020; Hiscox, Johnson, McGarry, Schwarb, et al., 2020; Study B = Schwarb, Johnson, McGarry, & Cohen, 2016; Schwarb et al., 2019, Johnson et al., 2016; Study C = Huesmann et al., 2020; Study D = Burzynska, Finc, Taylor, Knecht, & Kramer, 2017; Study E = Schwarb et al., 2017, Johnson et al., 2018.

### 2.3 MRE analysis

**FIGURE 1:**
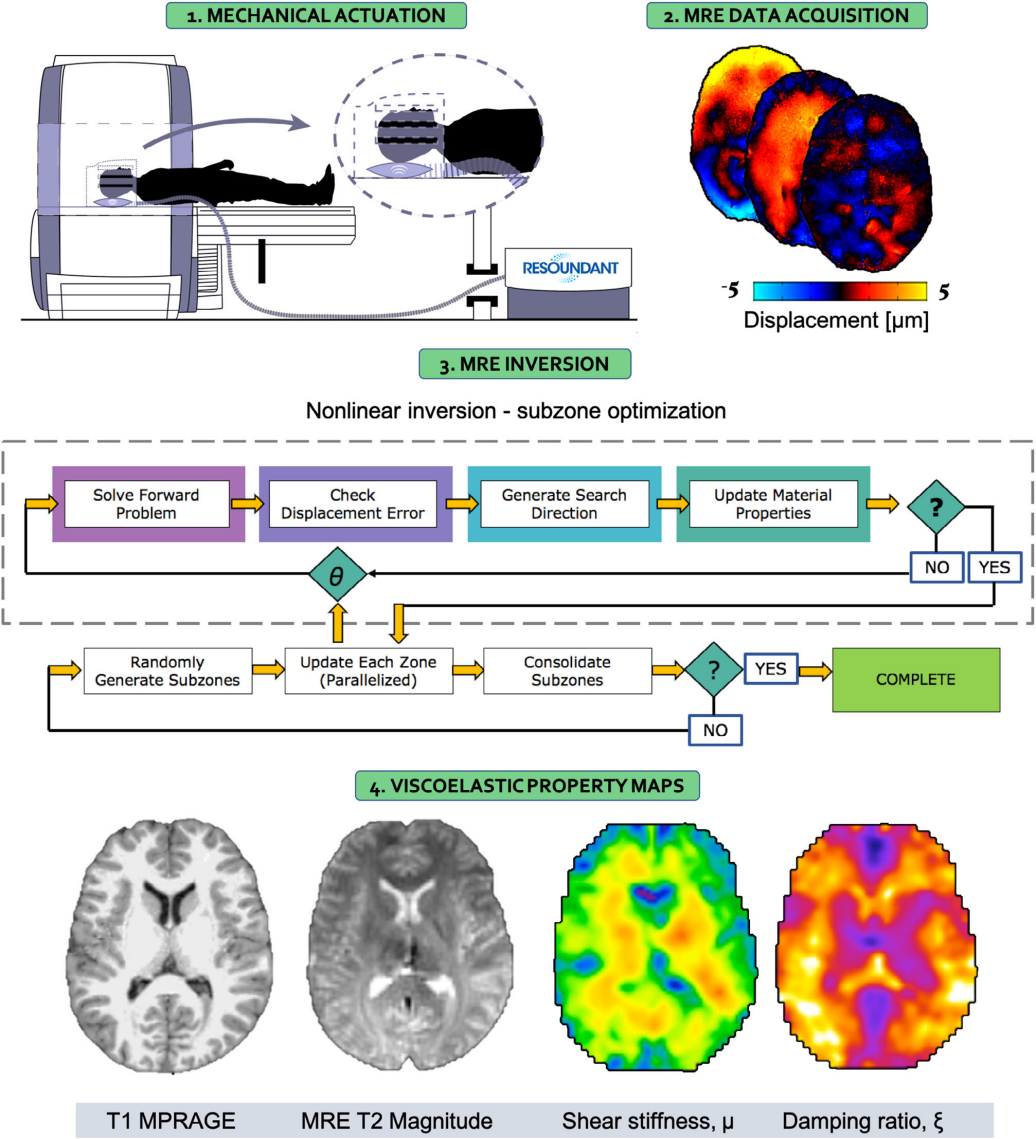
Overview of the MRE imaging and analysis procedure. In the first step, shear waves at 50 Hz are introduced to the brain via a pneumatic actuation system (Resoundant; Rochester, MN). The resulting tissue deformation is captured using motion‐encoding gradients embedded within the MRE spiral sequence, and displacement data is captured along three separate axes (anterior–posterior, right–left, and superior–inferior). The displacement data along with a binary brain mask is supplied to the nonlinear algorithm which models tissue as a heterogenous, viscoelastic material. A subzone optimization procedure is used to iteratively update the property description in a finite element computational model to minimize the difference between the model displacements and the measured displacement data. Finally, maps of the complex shear modulus are converted to shear stiffness, *μ* = 2|G*|2/(*G*' + |G*|), and damping ratio, ξ = *G*″/2*G*′. The subject specific T1‐weighted MPRAGE and MRE T2 magnitude images are provided to illustrate the images required for the spatial normalization procedure

MRE data for each subject met the required octahedral shear strain SNR (OSS‐SNR) threshold of 3 (McGarry et al., 2011), which is an accepted measure of brain MRE data quality. MRE displacement data was processed using nonlinear inversion (NLI) (McGarry et al., 2012; Van Houten, Paulsen, Miga, Kennedy, & Weaver, 1999). NLI applies a heterogenous viscoelastic finite element model to estimate the complex shear modulus, *G* ^*^ = *G′* + *iG″*, from the full vector MRE displacement data. The finite element property distribution is iteratively updated to match the model displacements to the measured displacement data using a subzone‐based optimization procedure. We used a 19.6 mm cubic subzone, which has been standardized and maintained across all published brain MRE studies from our group. To further maintain consistency with our previously published work, maps of the complex shear modulus G* were reformulated to provide quantitative maps of shear stiffness, *μ* = 2|*G* ^*^|^2^/(*G′*+|*G* ^*^|), and damping ratio, *ξ* = *G″*/2*G′*. Shear stiffness, *μ*, is a measure of the speed of the acoustic waves in a viscoelastic solid, with waves propagating faster in stiffer material. Stiffness measures have been reported to vary depending upon neuronal density and neurogenesis (Freimann et al., 2013; Klein et al., 2014), degree of myelination (Schregel et al., 2012; Weickenmeier et al., 2016; Weickenmeier, de Rooij, Budday, Ovaert, & Kuhl, 2017), and inflammation (Riek et al., 2012), and is the parameter most commonly reported to be affected in neurological disorders (Murphy et al., 2019). Damping ratio, *ξ*, is a dimensionless quantity that describes the relative displacement attenuation level in the material. Higher *ξ* values mean that shear wave oscillations attenuate more rapidly as they propagate suggesting that the tissue exhibits more viscous, fluid‐like behavior; in contrast, lower values indicate a more elastic‐solid material. The damping ratio, *ξ*, of the hippocampus has been linked to performance on memory tasks (Hiscox, Johnson, McGarry, Schwarb, et al., 2020; Schwarb et al., 2016, 2017), whereas *ξ* of the orbitofrontal cortex has been associated with fluid intelligence ability (Johnson et al., 2018). A flow diagram of the entire MRE processing pipeline is presented in Figure 1.

### 2.4 Spatial normalization

MRE T2‐weighted magnitude images and T1‐weighted images for each participant were skull stripped using the Brain Extraction Tool (BET) within the FMRIB Software Library (FSL) (Smith, 2002) and Freesurfer v6.0; (Fischl et al., 2002), respectively. MRE data were then registered to a common reference (MNI152 nonlinear T1‐weighted 2 mm brain atlas from the FSL database), within the spatial coordinates of the ICBM‐152 brain space (International Consortium for Brain Mapping), using Advanced Normalization Tools (ANTS) (Avants et al., 2011). First, the rigid body and affine transform from the MRE T2‐weighted magnitude image to the corresponding T1‐weighted image of the same participant was calculated using a mutual information similarity metric. Second, a nonlinear transform from the subject T1‐weighted image to the MNI152 template was calculated using a cross correlation standard symmetric normalization (SyN) transformation model with Gauss regularization [3,0] for diffeomorphic image registration. The number of iterations and number of resolution levels was set to 100 × 100 × 100 × 20. The affine transformation matrix and nonlinear warp were then applied simultaneously via concatenation to warp the MRE shear stiffness, *μ*, and damping ratio, *ξ*, images directly to the MNI152 template using linear interpolation. All images were inspected visually to assess correspondence to the target image. Output files were *μ* and *ξ* images in standard MNI space for each subject. Finally, *μ*_mean_ and *ξ*_mean_ atlases were created by averaging the normalized maps of all 134 participants. Dimensions of the normalized MRE templates were 91 × 109 × 91 voxels, and the final voxel‐size was 2 mm × 2 mm × 2 mm.

### 2.5 Masks of regions of interest

The process by which probabilistic masks were obtained in MNI space for each brain region of interest (ROI) is described below.

#### 2.5.1 Global masks

The whole brain mask used was the accompanying mask to the MNI152 T1‐weighted 2 mm atlas within FSL, whereas the white matter (WM) mask was generated by segmenting the MNI152 T1‐weighted template using Statistical Parametric Mapping software (SPM12 v7487, University College London, London, UK). The subcortical gray matter (SGM) mask was the combination of all subcortical regions investigated and the cortical gray matter (CGM) mask was the combination of all of the cortical regions investigated (see next sections).

#### 2.5.2 Subcortical gray matter

Six SGM ROIs were taken from the Mindboggle atlas (Klein et al., 2017) that uses complementary labelling protocols from the FreeSurfer aseg labels (Fischl et al., 2002). The six regions were amygdala (AM), caudate (CA), hippocampus (HC), pallidum (PA), putamen (PU), and thalamus (TH). All masks were extracted separately and then each eroded by 1 voxel to create more conservative representations of these structures and ensure greater confidence that reported MRE values were specific to the brain structure of interest. Final mask sizes were AM = 50; CA = 380; HC = 382; PA = 177; PU = 560; TH = 1,636 voxels.

#### 2.5.3 White matter tracts

A total of 12 major, long range, white matter tract masks were extracted; eight were obtained from the JHU‐ICBM‐tracts‐prob 2 mm atlas, and four were extracted from the JHU‐ICBM‐labels 2 mm atlas (corpus callosum, posterior thalamic radiation, corona radiata, and fornix). All probabilistic masks were thresholded at 20%. Masks included a selection of the three types of WM tracts—projection, commissural, and association. Projection tracts included corticospinal tract (CST), anterior thalamic radiation (ATR), posterior thalamic radiation (PTR), and corona radiata (CRa); commissural tracts included corpus callosum (CC), major forceps (FMa), minor forceps (FMi), and fornix (FX); association tracts included the uncinate fasciculus (UN), inferior frontal‐occipital fasciculus (IFOF), inferior longitudinal fasciculus (ILF), and superior longitudinal fasciculus (SLF). Mask sizes for each WMT ROI ranged between 81 voxels (fornix) up to 4,693 voxels (corpus callosum).

#### 2.5.4 Cortical gray matter

Twelve cortical ROIs were extracted from the Desikan–Killiany–Tourville cortical labelling protocol, which is also available from the Mindboggle atlas (Klein et al., 2017). ROIs were selected that were equally distributed across the brain (three regions each from the frontal, occipital, parietal, and temporal lobes), and were larger in size due to the limited spatial resolution. Frontal cortical regions include superior frontal cortex (SFC), rostral middle frontal (RMF), and precentral cortex (PRE); occipital regions were lateral occipital (LaO), lingual occipital (LiO), and cuneus (CN); parietal regions were superior parietal (SPC), postcentral (POST), and precuneus (PCN); and temporal regions included the superior temporal cortex (STC), inferior temporal cortex (ITC), and fusiform gyrus (FSG). Mask sizes for each cortical ROI ranged between 1,173 voxels (cuneus cortex) up to 8,010 voxels (superior frontal cortex).

### 2.6 Statistical analyses

Descriptive statistics for variables of interest are reported. General Linear mixed models were used to test the differences among regions for MRE measures of shear stiffness, *μ*, and damping ratio, *ξ* using an unstructured covariance matrix. Other fixed effects in the model included sex, and the sex by region of interest interaction to determine whether differences in viscoelastic properties among the individual regions differed according to sex. A study effect was included in all models to adjust for any differences found from combining data garnered from different projects. This study used a mixed model in lieu of the traditional ANOVA for two reasons. First, to directly specify the residual covariance matrix instead of trying to meet the assumption of sphericity. Second, the mixed model is able to produce parameter estimates with missing data in contrast to the listwise deletion inherent in an ANOVA. This allows for missing individual data points or instances, while still retaining the rest of that case's data. All model assumptions were tested, and if violated appropriate actions were taken to satisfy the assumptions. Normality was tested using the Shapiro–Wilk test (Ghasemi & Zahediasl, 2012). If normality was violated, model residuals were inspected using box‐plots and data points identified as outliers were removed. Significant model effects were followed up with *posthoc* pair‐wise comparisons using a Bonferroni correction to protect results from Type 1 errors. Statistically significant effects were determined at *p* < .05. All analyses were performed using the SPSS software version IBM SPSS Statistics for Mac, version 26.0.0 (IBM Corp., Armonk, NY).

## 3 RESULTS

**FIGURE 2:**
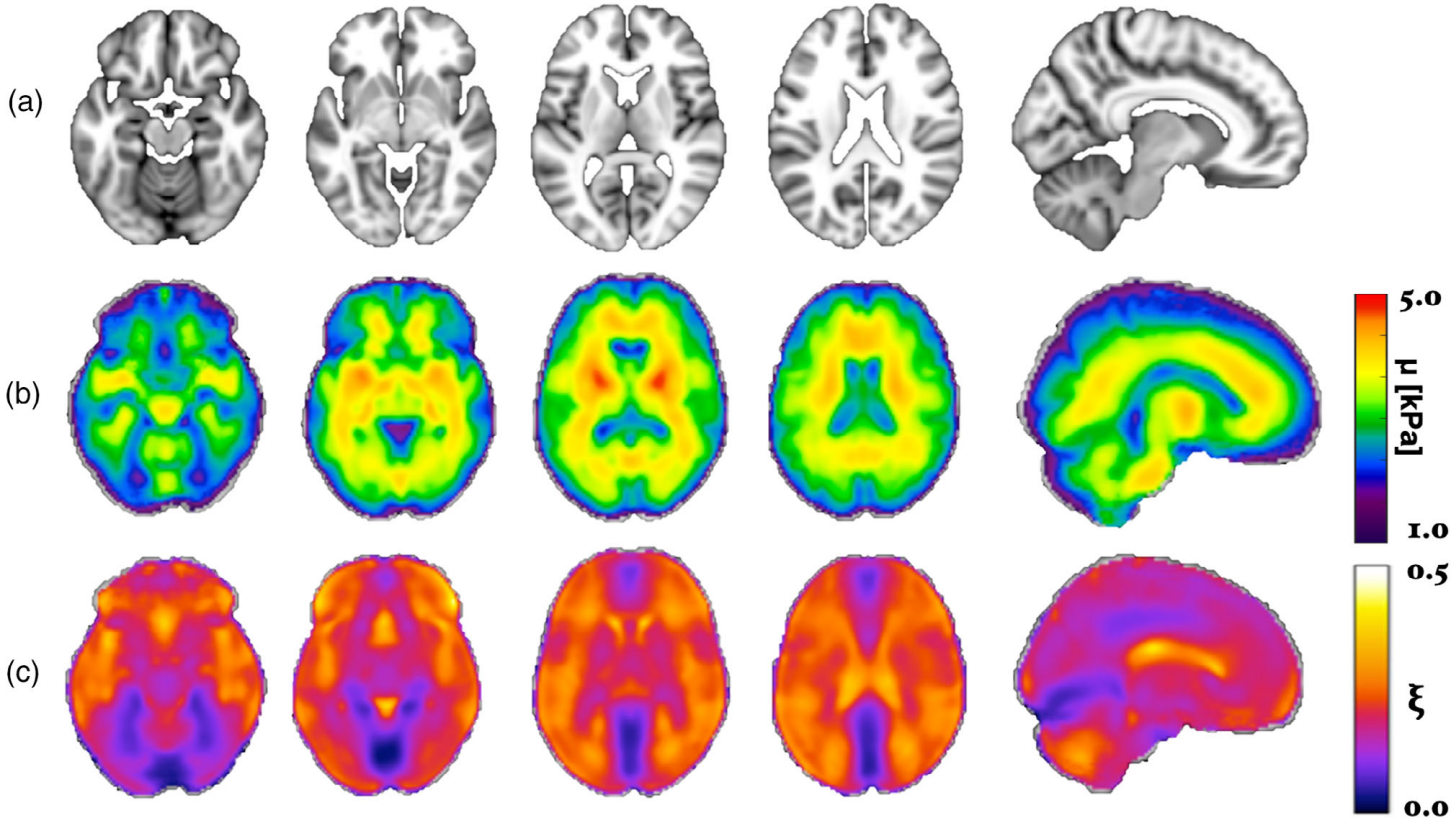
(a) Representative axial images and sagittal view (last column) from the MNI152 T1‐weighted template; (b) mean shear stiffness, *μ*_mean_, and (c) mean damping ratio, ξ_mean_, templates created by averaging the spatially normalized images from all 134 participants

MRE mean shear stiffness, *μ*_mean_, and damping ratio, *ξ*_mean_, atlases are shown in Figure 2. Visual comparison of the MRE templates to the MNI152 T1‐weighted template showed good correspondence of the size and location of brain structures between datasets. All subsequent results presented are determined in standard MNI space, though MRE measurements in native space are supplied in Supporting Information for reference.

### 3.1 Global regions of interest

**FIGURE 3:**
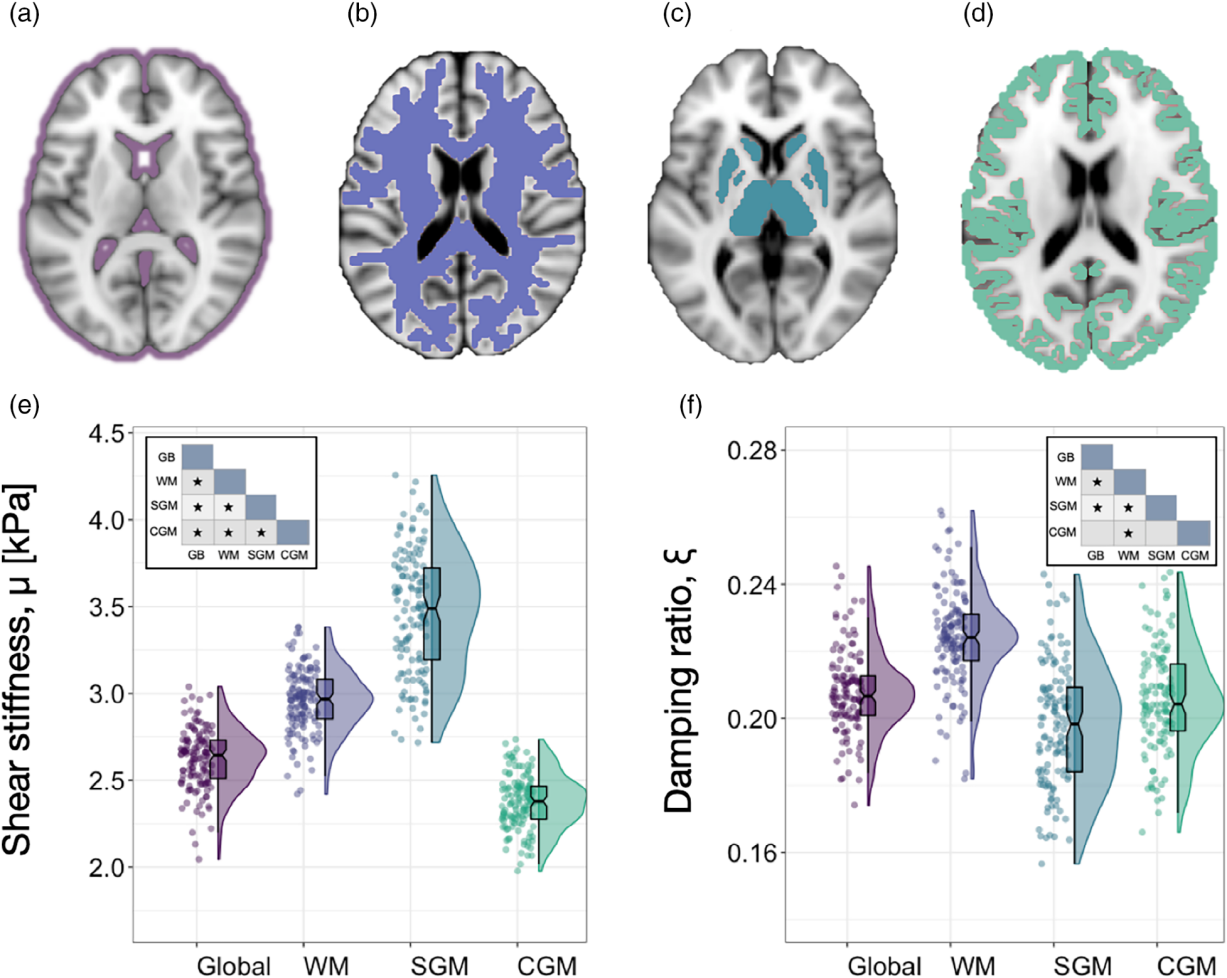
Panels (a–d) illustrate the binary masks used to quantify MRE measurements for (a) the entire brain excluding the ventricles, (b) white matter, (c) subcortical gray matter, and (d) the cerebral cortex. Note that panel (a) illustrates the excluded regions, whereas Panels (b–d) show the binary masks themselves. Variable density boxplots are provided for MRE measures of (e) shear stiffness, *μ*, and (f) damping ratio, ξ, for each global region of interest (ROI) to show data dispersion. The length of the box plots illustrates the 25th and 75th percentiles (i.e., interquartile range), with the central black line showing the median. Extended lines indicate the maximum and minimum values. Individual data points have been adjusted for study and sex by removing the relevant estimated coefficients from the mixed model. Inset shows Bonferroni corrected pairwise comparisons of each global ROI pair, * indicating *p* < .05

Descriptive statistics for MRE measures within each global ROI, based on modified population marginal means ± standard deviations, are provided in Table 2. Figure 3 shows the MRE global masks and distribution of data visualized through variable density boxplots.

**TABLE 2:** Descriptive statistics of MRE measures for each global ROI

	Shear stiffness, *μ* (kPa)			Damping ratio, *ξ*		
	Male	Female	Average	Male	Female	Average
Global	2.64 ± .19	2.60 ± .20	2.62 ± .21	.203 ± .021	.207 ± .020	.205 ± .024
WM	2.98 ± .21	2.93 ± .20	2.95 ± .21	**.217** ± .021*	**.225** ± .020*	.221 ± .024
SGM	3.48 ± .36	3.45 ± .36	3.46 ± .38	.197 ± .023	.203 ± .025	.200 ± .027
CGM	2.39 ± .19	2.35 ± .18	2.37 ± .18	.203 ± .025	.205 ± .025	.204 ± .027

*Note:* Mean + standard deviation (SD) are based on modified population marginal means supplied from the linear mixed model. Significant differences between males and females are indicated in bold and by *, *p* < .05.

Abbreviations: CGM, cortical gray matter; SGM subcortical gray matter; WM, white matter.

Shear stiffness, *μ* (kPa): Four outliers were identified for Global *μ*, four for WM, four for SGM, and four for CGM. A significant effect of region occurred, controlling for study and sex [*F*_(3,129)_ = 2,716, *p* < .001]; average global brain *μ* was 2.62 ± 0.17 kPa, WM was 2.95 ± 0.18 kPa, SGM was 3.46 ± 0.35 kPa, and CGM was 2.37 ± 0.16 kPa. Bonferroni pairwise comparisons indicated that all global ROIs were significantly different from one another (*p* < .001). No significant region × sex interaction was observed: [*F*_(3,129)_ = 0.45, *p* = .72].

Damping ratio, *ξ*: 10 outliers were identified for Global *ξ*, two for WM, three outliers for SGM, and seven for CGM. A significant effect of region was observed, controlling for study and sex. Regions were statistically significantly different [*F*_(3,122)_ = 400.67, *p* < .001]; average global *ξ* was 0.208 ± 0.015, WM was 0.225 ± 0.015, SGM was 0.200 ± 0.022, and CGM was 0.206 ± 0.019. Bonferroni pairwise comparisons indicated that WM *ξ* was significantly greater than the other three ROIs (all *p* < .001); Global *ξ* was also significantly higher than SGM *ξ* (*p* = .023).

A statistically significant interaction was also found between Global ROI *ξ* and sex: [*F*_(3,122)_ = 5.90, *p* = .001]; WM *ξ* was significantly greater in females (0.225 ± 0.020) compared to males (0.217 ± 0.021), (*p* = .021). No other pairwise comparison was significant (*p* > .05).

### 3.2 Subcortical gray matter

**FIGURE 4:**
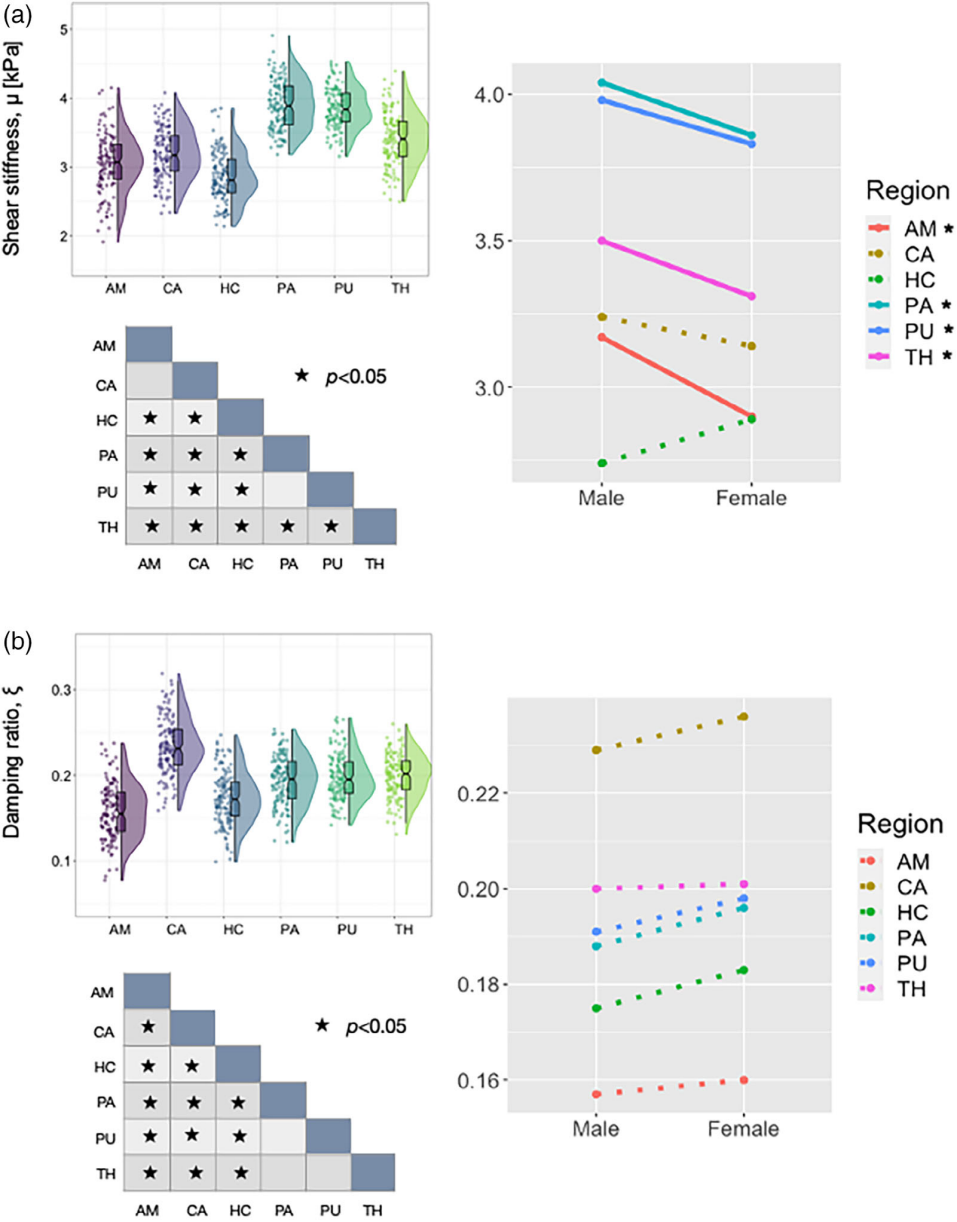
Variable density boxplots, significance charts, and sex x region interaction plots for subcortical gray matter (a) shear stiffness, *μ*, and (b) damping ratio, ξ. The length of the box plots illustrates the 25th and 75th percentiles (i.e., interquartile range), with the central black line showing the median. Individual data points have been adjusted for study and sex by removing the relevant estimated coefficients from the mixed model. Significant differences between structures were determined through post‐hoc linear correlations which were adjusted for multiple comparisons with Bonferroni correction. A significant interaction was found between sex and SGM, *μ*, with amygdala (AM; *p* = .024), pallidum (PA; *p* = .028), putamen (*p* = .031), and thalamus (TH; *p* = .018) being significantly stiffer in males. Hippocampus was the only SGM region stiffer in females (HC; *p* = .054). No significant sex differences were observed for *ξ* (*p* > .05)

Descriptive statistics based on modified population marginal means ± standard deviation is provided for SGM structures in Table 3. Figure 4 illustrates variable density boxplots, pairwise comparison tables, and sex × region interaction plots for SGM (a) shear stiffness, *μ*, and (b) damping ratio, *ξ*.

**TABLE 3:** Descriptive statistics of MRE measures for SGM ROIs

	Shear stiffness, *μ* (kPa)			Damping ratio, *ξ*		
	Male	Female	Average	Male	Female	Average
AM	3.17 ± .69*	2.90 ± .68*	3.04 ± .69	.157 ± .042	.160 ± .042	.158 ± .041
CA	3.24 ± .50	3.14 ± .47	3.19 ± .50	.229 ± .040	.236 ± .038	.232 ± .041
HC	2.74 ± .46	2.89 ± .45	2.82 ± .56	.175 ± .038	.183 ± .036	.179 ± .041
PA	**4.04** ± .48*	**3.86** ± .47*	3.95 ± .50	.188 ± .034	.196 ± .034	.192 ± .035
PU	3.98 ± .42*	3.83 ± .41*	3.91 ± .44	.191 ± .032	.198 ± .034	.195 ± .035
TH	3.50 ± .48*	3.31 ± .47*	3.41 ± .50	.200 ± .042	.201 ± .034	.200 ± .035

*Note:* Mean + standard deviation (SD) are based on modified population marginal means supplied from the linear mixed model. Significant differences between males and females are indicated in bold and by *, *p* < .05.

Abbreviations: AM, amygdala; CA, caudate; HC, hippocampus; PA, pallidum; PU, putamen; TH, thalamus.

Shear stiffness, *μ* (kPa): Five outliers were identified for AM, three for CA, six for HC, one for PA, three for PU, and two for TH. A significant effect of region on *μ* was observed [*F*_(5,126)_ = 197.71, *p* < .001]. Mean stiffness for AM was 3.04 ± 0.69 kPa, CA was 3.19 ± 0.50 kPa, HC was 2.82 ± 0.56 kPa, PA was 3.95 ± 0.50 kPa, PU was 3.91 ± 0.44 kPa, and TH was 3.41 ± 0.50 kPa. Bonferroni pairwise comparisons indicated that a large proportion of regions differed in stiffness, as indicated in the pairwise comparison table in Figure 4a. In particular, PA and PU were significantly stiffer than all other SGM ROIs (*p* < .001), and AM and CA did not differ from one another (*p* = .36).

A statistically significant interaction was also found between SGM *μ* and sex: [*F*_(5,126)_ = 6.38, *p* < .001]. AM (*p* = .024), PA (*p* = .028), PU (*p* = .031), and TH (*p* = .018) were significantly stiffer in males compared to females. In contrast, HC was stiffer in females, yet did not reach the threshold for statistical significance (*p* = .054).

Damping ratio, *ξ*: Four outliers were identified for AM, one for CA, one for HC, three for PA, three for PU, and two for TH. A significant effect of region on *ξ* was observed [*F*_(5,128)_ = 70.49, *p* < .001]. Mean *ξ* for AM was 0.158 ± 0.041, CA was 0.232 ± 0.041, HC was 0.179 ± 0.041, PA was 0.192 ± 0.035, PU was 0.195 ± 0.035, and TH was 0.200 ± 0.035. Bonferroni pairwise comparisons showed that the majority of SGM differed in *ξ*, as illustrated in Figure 4b. CA had highest *ξ* and was significantly greater than all other regions. Lowest *ξ* was found for AM and this measure was significantly lower when compared to any other region. No significant region × sex interaction was observed [*F*_(5,128)_ = 0.70, *p* = .62].

### 3.3 White matter tracts

**FIGURE 5:**
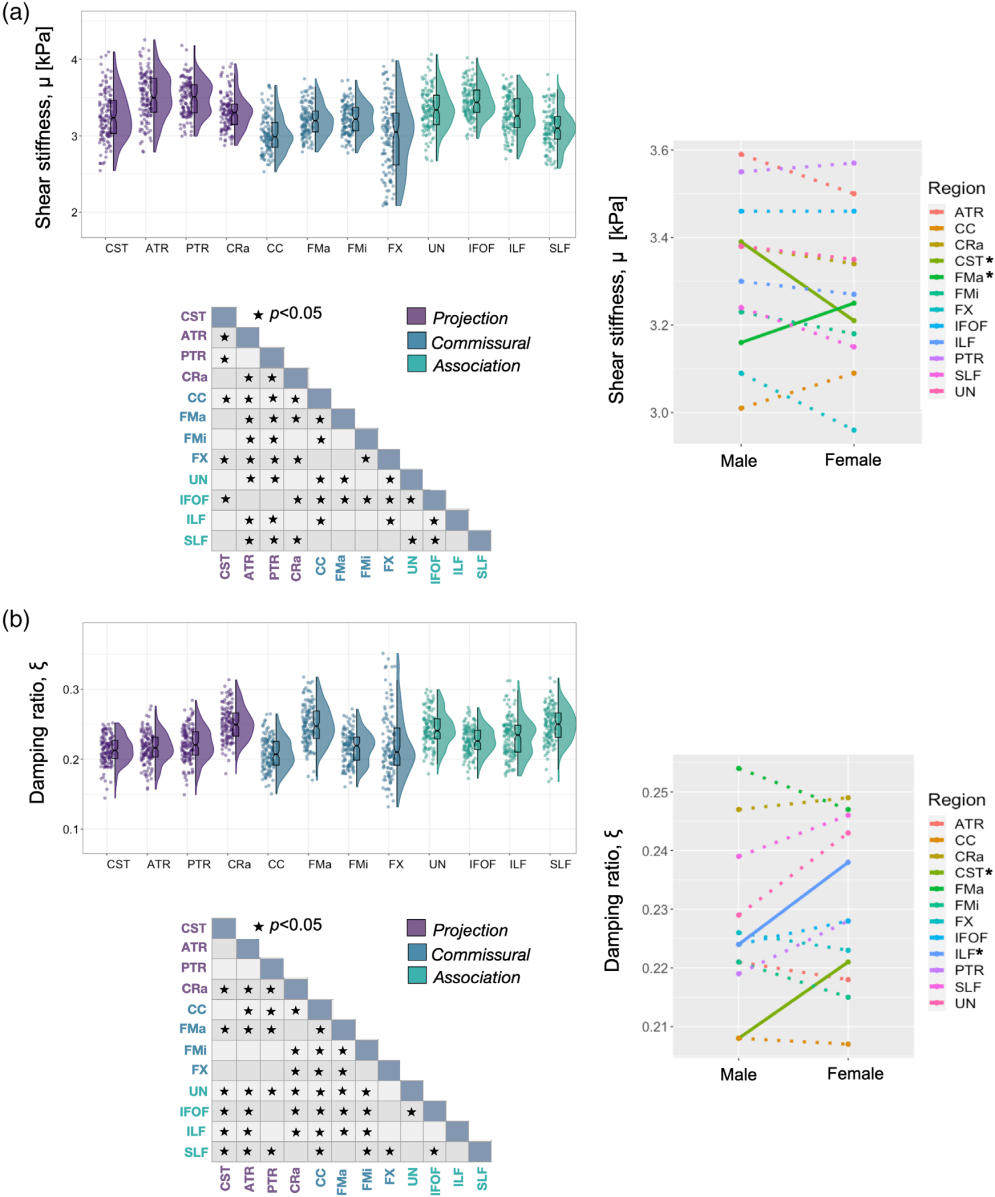
Variable density boxplots, pairwise significant charts, and sex x region interaction plots for white matter tract (a) shear stiffness, *μ*, and (b) damping ratio, *ξ*. The length of the box plots illustrates the 25th and 75th percentiles (i.e., interquartile range), with the central black line showing the median. Extended lines indicate the maximum and minimum values. Individual data points have been adjusted for study and sex by removing the relevant estimated coefficients from the mixed model. Significant differences between structures were determined through post‐hoc linear correlations which were adjusted for multiple comparisons with Bonferroni correction. A significant interaction was found between sex and WMT *μ*, with corticospinal tract (CST; *p* = .007) being stiffer in males. In contrast, the major forceps (FMa; *p* = .041) were significantly stiffer in females. For *ξ*, females had greater *ξ* in both the corticospinal tract (CST; *p* = .005), and inferior longitudinal fasciculus (ILF; *p* = .020). No other pairwise comparison was significant for either measure (*p* > .05)

Descriptive statistics based on modified population marginal means ± standard deviations for WMTs are provided in Table 4. Figure 5 illustrates variable density boxplots, pairwise comparison tables, and sex × region interaction plots for WMT (a) shear stiffness, *μ*, and (b) damping ratio, *ξ*.

**TABLE 4:** Descriptive statistics of MRE measures for WMT ROIs

	Shear stiffness, *μ* (kPa)			Damping ratio, *ξ*		
	Male	Female	Average	Male	Female	Average
*Projection tracts*						
CST	**3.39** ± .38*	**3.21** ± .38*	3.30 ± .39	**.208** ± .029*	**.221** ± .027*	.214 ± .030
ATR	3.59 ± .37	3.50 ± .37	3.54 ± .38	.221 ± .029	.218 ± .027	.220 ± .027
PTR	3.55 ± .33	3.57 ± .32	3.56 ± .34	.219 ± .011	.228 ± .029	.223 ± .032
CRa	3.38 ± .32	3.34 ± .32	3.36 ± .33	.247 ± .031	.249 ± .032	.248 ± .030
*Commissural tracts*						
CC	3.01 ± .31	3.09 ± .31	3.05 ± .32	.208 ± .027	.207 ± .027	.208 ± .027
FMa	**3.16** ± .27*	**3.25** ± .27*	3.21 ± .28	.254 ± .034	.247 ± .034	.250 ± .032
FMi	3.23 ± .26	3.18 ± .26	3.21 ± .27	.221 ± .029	.215 ± .029	.218 ± .030
FX	3.09 ± .56	2.96 ± .55	3.02 ± .56	.226 ± .053	.223 ± .052	.224 ± .056
*Association tracts*						
UN	3.38 ± .34	3.35 ± .33	3.36 ± .35	.229 ± .040	.243 ± .041	.236 ± .041
IFOF	3.46 ± .28	3.46 ± .28	3.46 ± .29	.224 ± .027	.228 ± .027	.226 ± .027
ILF	3.30 ± .33	3.27 ± .33	3.29 ± .34	**.224** ± .036*	**.238** ± .034*	.231 ± .035
SLF	3.24 ± .34	3.15 ± .34	3.19 ± .35	.239 ± .032	.246 ± .034	.243 ± .032

*Note:* Mean + standard deviation (SD) based on modified population marginal means supplied from the linear mixed model. Significant differences between males and females are indicated in bold and by *, *p* < .05.

Abbreviations: CST, corticospinal tract; ATR anterior thalamic radiation; PTR, posterior thalamic radiation; CRa, corona radiata; CC, corpus callosum; FMa, major forceps; FMi, minor forceps; FX, fornix; UN, uncinate; IFOF, inferior frontal‐occipital fasciculus; ILF, inferior longitudinal fasciculus; SLF, superior longitudinal fasciculus.

Shear stiffness, *μ* (kPa): Outliers were identified for 12 WMT regions, with a minimum of one and maximum of seven outliers per region. A significant effect of region on *μ* was found [*F*_(11,129)_ = 117, *p* < .001]. Pairwise comparisons showed that a large proportion of regions were significantly different from one another; PTR and ATR exhibited highest stiffness, (3.56 ± 0.34 kPa and 3.54 ± 0.38 kPa, respectively), whereas lowest stiffness was observed in FX (3.02 ± 0.56 kPa), which was significantly softer when compared to all other ROIs, except for CC (3.05 ± 0.32), as shown in the pairwise comparison table in Figure 5a.

A statistically significant interaction was found between WMT *μ* and sex [*F*_(11,129)_ = 4.23, *p* < .001]. CST was stiffer in males (*p* = .007), whereas FMa was stiffer in females (*p* = .041). No other pairwise comparison was significant (*p* > .05).

Damping ratio, *ξ*: Outliers were identified for 12 WMT regions, with a minimum of zero and maximum of five outliers per region. A significant effect of region existed on *ξ* [*F*_(11,128)_ = 90.10, *p* < .001]. FMaj exhibited greatest *ξ* (0.250 ± 0.032) and was significantly greater that all other WMTs except for CRa and SLF, whereas lowest *ξ* was observed in CC (0.208 ± 0.027) but did not differ when compared to CST (0.214 ± 0.030). Pairwise comparisons showed that a large proportion of regions were significantly different from one another, as shown in the pairwise comparison table in Figure 5b.

A statistically significant interaction was also found between WMT *ξ* and sex [*F*_(11,128)_ = 3.38, *p* < .001]. CST and ILF *ξ* were both higher in females (*p* = .005, *p* = .020, respectively). No other pairwise comparison was significant (*p* > .05).

### 3.4 Cortical gray matter

**FIGURE 6:**
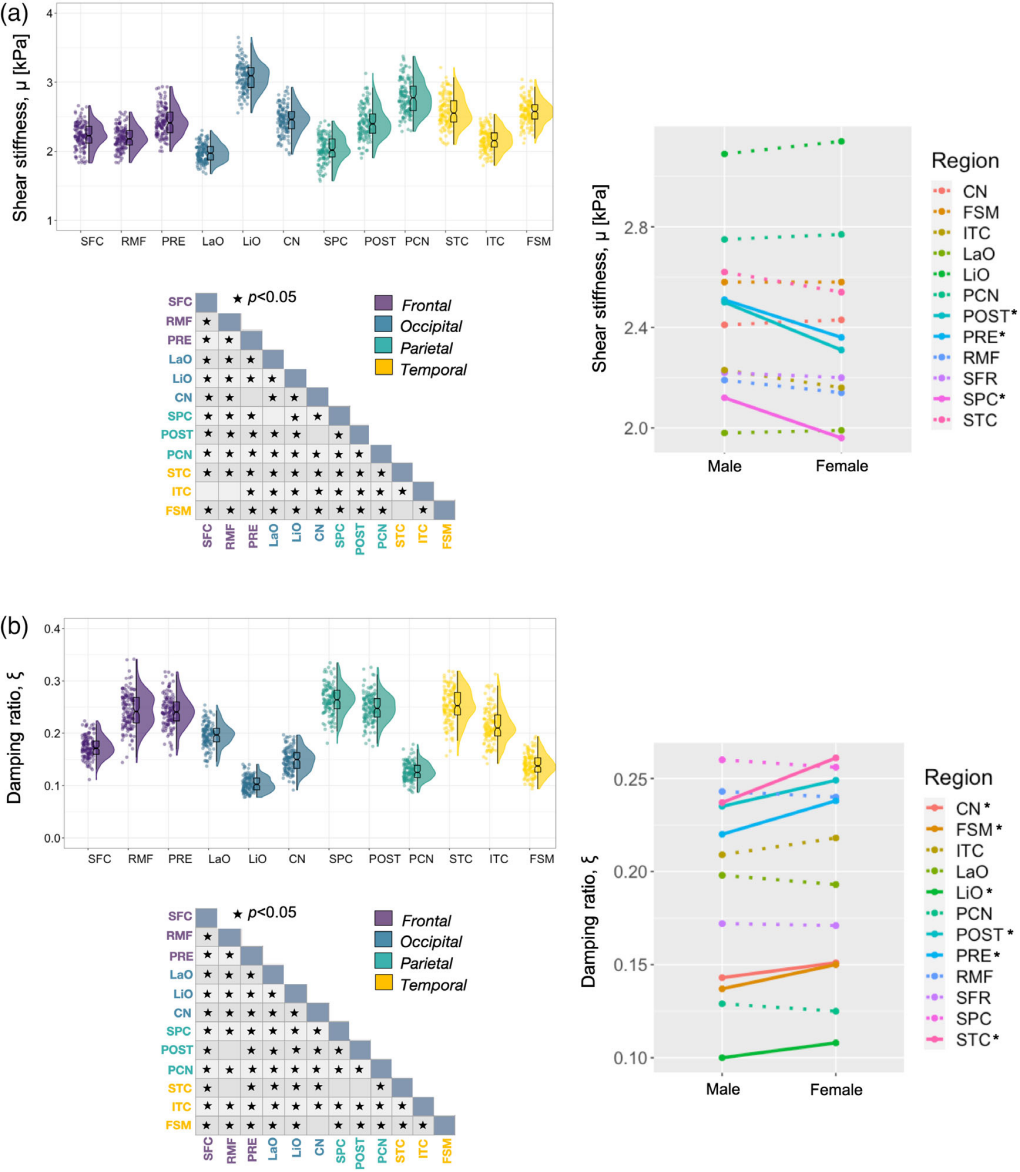
Variable density boxplots, pairwise significant charts, and sex x region interaction plots for cortical gray matter (a) shear stiffness, *μ*, and (b) damping ratio, *ξ*. The length of the box plots illustrates the 25th and 75th percentiles (i.e., interquartile range), with the central black line showing the median. Extended lines indicate the maximum and minimum values. Individual data points have been adjusted for study and sex by removing the relevant estimated coefficients from the mixed model. Significant differences between structures were determined through post‐hoc linear correlations which were adjusted for multiple comparisons with Bonferroni correction. A significant interaction was found between sex and CGM *μ*, with the postcentral cortex (POST; *p* < .001), precuneus (PCN; *p* < .001), and superior parietal cortex (SPC; *p* < .001) being stiffer in males. A significant interaction was also found for *ξ*; females had greater *ξ* for cuneus (CN; *p* = .046), fusiform (FSM; *p* = .007), lingual occipital (LiO; *p* = .010), precentral (PRE; *p* = .014), postcentral (POST; *p* = .025), and superior temporal (STC; *p* = .005) cortices

Descriptive statistics based on modified population marginal means ± standard deviation for both MRE measures for CGM are provided in Table 5. Figure 6 illustrates variable density boxplots, pairwise comparison tables, and sex x region interaction plots for CGM (a) shear stiffness, *μ*, and (b) damping ratio, *ξ*.

**TABLE 5:** Descriptive statistics of MRE measures for CGM ROIs

	Shear stiffness, *μ* (kPa)			Damping ratio, *ξ*		
	Male	Female	Average	Male	Female	Average
*Frontal lobe*						
SFC	2.22 ± .22	2.20 ± .22	2.21 ± .22	.172 ± .025	.171 ± .025	.172 ± .027
RMF	2.19 ± .20	2.14 ± .20	2.16 ± .21	.243 ± .036	.240 ± .037	.242 ± .038
PRE	2.51 ± .25*	2.36 ± .25*	2.44 ± .26	.220 ± .042*	.238 ± .041*	.229 ± .041
*Occipital lobe*						
LaO	1.98 ± .17	1.99 ± .17	1.99 ± .18	.198 ± .029	.193 ± .027	.196 ± .027
LiO	3.09 ± .26	3.14 ± .25	3.12 ± .26	.100 ± .017*	.108 ± .020*	.104 ± .021
CN	2.41 ± .24	2.43 ± .24	2.42 ± .25	.143 ± .023*	.151 ± .025*	.147 ± .024
*Parietal lobe*						
SPC	2.12 ± .27*	1.96 ± .27*	2.04 ± .27	.260 ± .036	.256 ± .038	.258 ± .038
POST	2.50 ± .26*	2.31 ± .26*	2.40 ± .27	.235 ± .036*	.249 ± .036*	.242 ± .035
PCN	2.75 ± .31	2.77 ± .31	2.76 ± .32	.129 ± .021	.125 ± .020	.127 ± .021
*Temporal lobe*						
STC	2.62 ± .25	2.54 ± .25	2.58 ± .26	.237 ± .044*	.261 ± .043*	.249 ± .044
ITC	2.23 ± .20	2.16 ± .20	2.20 ± .21	.209 ± .044	.218 ± .043	.213 ± .044
FSM	2.58 ± .23	2.58 ± .23	2.58 ± .24	.137 ± .029*	.150 ± .027*	.144 ± .030

*Note:* Mean + standard deviation (SD) based on modified population marginal means supplied from the linear mixed model. Significant differences between males and females are indicated in bold and by *, *p* < .05.

Abbreviations: CN, cuneus; FSM, fusiform; ITC, inferior temporal; LaO, lateral occipital; LiO, lingual occipital; PCN, precuneus; PRE, precentral; POST, postcentral; RMF, rostral middle frontal; SFC, superior frontal; SPC, superior parietal; STC, superior temporal.

Shear stiffness, *μ* (kPa): Outliers were identified for 12 CGM regions, with a minimum of zero and maximum of five outliers per region. There was a significant effect of region on *μ* [*F*_(11,129)_ = 488.25, *p* < .001]. Lingual occipital cortex exhibited highest stiffness (3.12 ± 0.26 kPa), whereas lowest stiffness was observed in the lateral occipital cortex (1.99 ± 0.18 kPa). Pairwise comparisons showed that a large proportion of regions were significantly different from one another, as shown in the pairwise comparison table in Figure 6a.

A statistically significant interaction was also found between cortical *μ* and sex [*F*_(11,129)_ = 6.85, *p* < .001]. Sex differences were revealed in the postcentral (*p* < .001), precentral (*p* < .001), and superior parietal cortex (*p* < .001). All exhibited higher *μ* in males. No other cortical ROI sex comparison was significant (*p* > .05).

Damping ratio, *ξ*: Outliers were identified for 12 CGM regions, with a minimum of two and maximum of three outliers per region. There was a significant effect of region on *ξ* [*F*_(11,130)_ = 288.12, *p* < .001]. Superior parietal cortex showed highest *ξ* (0.258 ± 0.038), whereas lowest *ξ* was reported for the lingual occipital cortex (0.104 ± 0.021). Again, pairwise comparisons showed that a large proportion of regions were significantly different from one another, as shown in the pairwise comparison table in Figure 6b.

A statistically significant interaction was also found between cortical *ξ* and sex [*F*_(11,130)_ = 5.28, *p* < .001]. Sex differences were revealed in cuneus (*p* = .046), fusiform (*p* = .007), lingual occipital (*p* = .010), precentral (*p* = .014), postcentral (*p* = .025), and superior temporal (*p* = .001) cortices; all exhibited higher *ξ* in females. No other cortical ROI sex comparison was significant (*p* > .05).

## 4 DISCUSSION

In this work, an in vivo atlas of the mechanical properties of the human brain has been created from a healthy young adult population. Using advanced nonlinear registration methods, MRE data were spatially normalized to a standard structural T1‐weighted image in MNI space to reveal new insights into the distribution of viscoelastic mechanical properties throughout the brain. To complement this atlas, the viscoelastic property measures and variation of a wide range of brain structures including global regions of interest, subcortical gray matter (SGM), white matter tracts (WMT), and cortical gray matter (CGM) are reported. Notably, we show that the majority of brain structures exhibit local mechanical properties that are distinct, variable, and are likely due to differences in neuronal tissue composition and organization. The observed general high variability of each measure across the population is likely to reflect individual differences in brain tissue microstructure that give rise to these properties. Notably, a large proportion of neuroanatomical structures were found to differ in their viscoelastic characteristics between males and females.

Considering the global measures, on average the brain has a shear stiffness of approximately 2.6 kPa; thus, the brain is one of the softest organs in the human body and softer than skeletal muscle (Chakouch, Charleux, & Bensamoun, 2015), heart (Khan, Fakhouri, Majeed, & Kolipaka, 2018), and kidneys (Gandhi et al., 2019). We report that global WM is approximately 20% stiffer than CGM: 2.95 ± 0.21 kPa versus 2.37 ± 0.18 kPa, respectively, which is in agreement with the existing MRE literature from several separate MRE research groups (Braun et al., 2014; Johnson et al., 2013; Zhang, Green, Sinkus, & Bilston, 2011). These results also support some investigations conducted at the tissue level; a study that used microindentation on freshly resected, human brain tissue also reported that WM was stiffer than cortical GM when evaluated with various strain rates and relaxation function parameters (Finan, Sundaresh, Elkin, McKhann, & Morrison, 2017). Mechanical indentation tests have also revealed that GM is approximately one third softer than WM in the porcine (Kaster, Sack, & Samani, 2011; van Dommelen, van der Sande, Hrapko, & Peters, 2010) and bovine brain (Budday et al., 2015), although other studies from the microindentation literature have shown the opposite trend (Budday et al., 2017; Park, Lonsberry, Gearing, Levey, & Desai, 2019). The discrepancy is possibly due to investigations being performed at the microscale returning mechanical properties that differ from the macroscale properties measured with MRE. For example, at the single cell level GM is likely to be stiffer than WM because it contains more (stiff) neuronal cell bodies (Lu et al., 2006), while macroscale WM measures are likely to be stiffer because of the structural organization of highly aligned axons. The difference in the rate of deformation between the two methods may also be important: for example, the harmonic motion of MRE applied at 50 Hz will possibly return different relative properties between structures as compared to measurements from quasi‐static indentations used in ex vivo studies. Other factors that may lead to differences between in vivo human brain MRE measurements and ex vivo animal experiments should be considered. For example, using MRE in the same animals at overlapping frequencies, measurements of porcine brain tissue in vivo were stiffer than porcine brain tissue samples measured ex vivo (Guertler et al., 2018). Higher modulus observed in the living human brain may be attributed to the confining nature of the skull creating a stiffening artifact (Gefen & Margulies, 2004), to blood flow and arterial tension (Hatt, Cheng, Tan, Sinkus, & Bilston, 2015; Hetzer et al., 2017), or raised intracranial pressure (Arani et al., 2018). There may also be inherent differences in the mechanical tissue structure of the human brain compared with those of other animals (Nicolle, Lounis, & Willinger, 2004; Prange & Margulies, 2002).

SGM is approximately 15% stiffer than WM and 35% stiffer than CGM, with minimal overlap occurring between SGM and CGM stiffness across all participants (95% CI: 3.40–3.53 kPa and 2.34–2.40 kPa, respectively). SGM may be significantly stiffer than CGM (3.40 kPa vs. 2.38 kPa) because subcortical structures possess a denser cell structure through more aligned fibers and greater myelination as revealed through histology and other MRI techniques such as myelin volume fraction imaging (Hagiwara et al., 2018). The specific geometry of both gyri and sulci may also influence CGM estimates as the lower resolution finite‐element mesh used in NLI treats cortical folds as a continuum. Due to the regularization process required to ensure measurement stability, the small‐scale, stiff‐soft‐stiff transitions across sulcal boundaries may be better represented through high‐resolution custom meshes to include the texture of the cortex.

The pallidum and putamen exhibit considerably higher stiffness compared to other SGM structures; a finding which has been consistently observed across MRE studies from separate research groups (Hetzer et al., 2017; Hiscox et al., 2018; Johnson et al., 2016). Our results indicate that the pallidum is 40% stiffer than the cerebrum implying a unique tissue composition that could be due to its highly unusual ultrastructure. Not only does the pallidum consist of a repetitive geometric arrangement of dendrites that are completely covered by axon terminals (Difiglia, Pasik, & Pasik, 1982), but it also strongly accumulates metalloprotein‐bound iron, which has been reflected in variations to diffusion tensor values (Syka et al., 2015), although it is not clear whether iron content is associated with viscoelasticity measures. Results indicate both structures also possess similar measures for damping ratio suggesting that the cytoarchitecture between the two regions are very similar. Some evidence suggests that, at least for the putamen, higher stiffness may be related to greater cerebral blood flow (Hetzer et al., 2017), with the putamen receiving its vascular supply directly from the middle and anterior cerebral arteries.

All of the major WMTs investigated were determined to be stiffer than global WM; with global WM including measures of both superficial and minor WMTs. Several studies have reported that the corona radiata is stiffer than the corpus callosum (Budday et al., 2017; Johnson et al., 2013), which agrees with our measurements of 3.36 ± 0.33 kPa and 3.05 ± 0.32 kPa, respectively. There is evidence to suggest that the corona radiata possesses more myelin than the corpus callosum (Chopra et al., 2018), and thus myelin content could contribute to the higher stiffness reported (Weickenmeier et al., 2016, 2017). Notably, the fornix, which is part of the limbic system and is critically involved in the formation of new memories (Douet & Chang, 2015; Schwarb et al., 2019), is particularly soft and exhibits greatest variability among participants for both MRE measures. The large variability reported here may suggest that fornix viscoelasticity could be promising for detecting individual differences that may relate to functional outcomes, especially as its integrity has been implicated in the transition from mild cognitive impairment to Alzheimer's disease (Nowrangi & Rosenberg, 2015; Oishi, Mielke, Albert, Lyketsos, & Mori, 2012). Given its close proximity to the lateral ventricles, however, partial volume effects with CSF may occur. In fact, high variability is generally observed across all WMTs which may perhaps reflect more noise and less reliable measurements. Future work could therefore consider mechanical heterogeneity within major tracts that result from multiple fiber pathways (Johnson et al., 2013), as well as more advanced anisotropic mechanical models as the backbone of the inversion algorithm (Schmidt et al., 2018; Smith et al., 2020) to minimize data‐model mismatch from incorrectly assuming that the WMTs are mechanically isotropic, that is, that mechanical properties at a given point are the same in all directions.

This work represents the first detailed investigation of the mechanical properties of parcellations of the cerebral cortex, though initial MRE investigations into cortical structure–function relationships (Johnson et al., 2018; Schwarb et al., 2019), contributions to adolescent risk‐taking behavior (McIlvain et al., 2020), and the mechanical integrity of the cortex in Alzheimer's disease (Hiscox, Johnson, McGarry, Marshall, et al., 2020) have been reported. We show that separate regions of the cortex exhibit different viscoelastic properties and, remarkably in some cases, observe distinct viscoelastic properties which do not overlap in the range of values across participants. For example, the individual stiffness measures for the lingual occipital cortex (3.12 ± 0.26 kPa), did not overlap with any measures obtained for the lateral occipital cortex (1.99 ± 0.18 kPa), even though these regions both reside in the occipital lobe. This lends strong support to suggest that cortical regions are distinct in their viscoelasticity, which therefore implies, they are unique in their neuronal architecture.

This MRE study is the first to report significant sex differences in viscoelasticity in a wide range of neuroanatomical structures which supplements the wealth of existing data that reports sex differences in neuroanatomy. Interestingly, we found that female brains are approximately 4% more viscous compared to males as indicated by significantly higher *ξ* in global WM, which contradicts an early study that reported female brains were 9% less viscous in large regions primarily comprising white matter (Sack, Streitberger, Krefting, Paul, & Braun, 2011). The protocols used in each study are substantially different and thus it is difficult to draw conclusions from this discrepancy. However, in the current study, we can identify and localize the effect of higher damping ratio in females within both the corticospinal tract (CST) and inferior longitudinal fasciculus (ILF) that would encompass a large volume of white matter. Sex differences in axonal shape, average area, and diameter (Zhou, Goto, Goto, Moriyama, & He, 2000) and measures of fractional anisotropy from diffusion imaging (Jung et al., 2019) have been reported in the CST, which may give rise to the findings reported here, including the higher stiffness of the CST reported in males.

The majority of subcortical structures exhibited sexual dimorphisms with the amygdala, pallidum, putamen, and thalamus all being significantly stiffer in males than females. These results suggest fundamental differences in tissue microstructure between the sexes in deep brain regions which warrant further investigation into how differences in viscoelasticity may relate to functional outcomes and behavior. Of all the SGM structures, only the stiffness of the hippocampus was higher in females than males, although this finding did not quite reach statistical significance (*p* = .054). Nonetheless, sex differences in specific domains of memory performance are well documented (Asperholm, Högman, Rafi, & Herlitz, 2019) and animal models have shown that females have increased spine density in the hippocampus due to sex‐specific signaling mechanisms (Hyer, Phillips, & Neigh, 2018). This outcome may affect structure–function relationships between hippocampal viscoelasticity and memory performance recently reported (Hiscox, Johnson, McGarry, Schwarb, et al., 2020; Johnson et al., 2018; Schwarb et al., 2016, 2017), and further investigations into the role of sex in these relationships are warranted.

Sex differences in cortical cytoarchitecture have also been reported (Rabinowicz et al., 2002), and the data presented here support the conclusions that fundamental sex differences exist in the structure of the cerebral cortex. In the current study, males were observed to possess significantly stiffer cortical gray matter within the frontal and parietal lobes, including the precentral, postcentral, and superior parietal cortices. These results are consistent with a previous microindentation investigation performed on freshly dissected human tissue (Finan et al., 2017), which illustrated that males possessed substantially stiffer tissue compared to females within the cortex. These observations may be relevant to reports that female athletes experience more frequent and severe concussions than males (Ono et al., 2015) as softer tissue may deform more easily during impact. There is some evidence to suggest that these relationships may evolve over time, as a previous MRE study reported how the temporal and occipital lobes were stiffer in older adult women (Arani et al., 2015), and therefore, further work is needed to elucidate how advancing age may change the sexual dimorphisms reported here. For damping ratio, we found that the majority of cortical regions are higher in females with, in one example, the superior temporal gyrus being 10% more viscous in women. Females have been shown to possess greater gyral complexity which reflects more sulcal bifurcations and cortical convolutions (Herron, Kang, & Woods, 2015) as well as significantly larger neuropil volumes than males (Rabinowicz et al., 2002). These may be possible candidate in vivo markers for damping ratio measures, which will require further study.

Our detailed measurements of brain viscoelasticity may have important clinical applications; for example, in aiding predictions of circumstances that lead to brain injuries after trauma, with acute neuroinflammation (Fehlner et al., 2016; Riek et al., 2012) and edema (Boulet, Kelso, & Othman, 2013) having been previously associated with brain tissue viscoelasticity. Accurate mechanical models are being sought to predict the degree of intracranial deformation occurring as a result of head impact (i.e., trauma) across a range of time scales and impact conditions (Zhao, Choate, & Ji, 2018), and due to the coup and contrecoup phenomenon, the cortex may be particularly vulnerable at the point of impact. While ex vivo studies of brain specimens have provided a wealth of important information in this regard, brain tissue degrades quickly (Zhang, Wu, et al., 2018; Zhang, Liu, et al., ), becomes stiffer over time (Garo, Hrapko, van Dommelen, & Peters, 2007), and may not recapitulate important processes that occur in vivo that may, in fact, couple neurophysiology to mechanical stiffness (Chatelin et al., 2016). In the same context, the general conditions that underly MRE investigations also have inherent limitations. For example, as brain tissue exhibits frequency‐dependent (Clayton, Garbow, & Bayly, 2011; Klatt, Hamhaber, Asbach, Braun, & Sack, 2007) and nonlinear behavior (Budday, Ovaert, Holzapfel, Steinmann, & Kuhl, 2019), the observed properties will directly depend on the frequency of excitation due to the frequency dependence of the stress–strain phenomena. For this study, this suggests that estimates of viscoelasticity are uniquely valid for 50 Hz deformations. Nevertheless, the reported mechanical properties reflect important features of the brain's composition and behavior, and the relative differences in viscoelasticity between brain structures and between sexes will have important clinical implications for TBI modeling (Barbey et al., 2015), and the development of simulations for neurosurgical techniques (Miller et al., 2019).

Although in this study we utilize 1.6 and 2.0 mm isotropic MRE data to build the viscoelastic template, which are considered high‐resolution for brain MRE, insufficient resolution may affect the accuracy of measurements in some of the smallest brain structures (Johnson et al., 2014, 2016). As such, we chose not to analyze regions such as individual nuclei and subfields of subcortical gray matter structures as well as some of the smaller cortical gray matter structures. Further, local SNR differences between structures and subjects may affect our results, though such local effects have not been previously established, and we have followed standard practice by ensuring global OSS‐SNR met the required threshold for stable inversion. Future work utilizing simulation and phantom experiments may consider how local SNR in smaller regions of interest could impact regional MRE measurements. Furthermore, the wavelength of shear waves provided from 50 Hz actuation may also limit the attainable resolution as higher vibration frequencies with shorter wavelengths could theoretically provide higher spatial resolutions. As is the case with all MRE investigations, the choice of frequency involves balancing the tradeoff between depth of penetration, shear wavelength, and noise levels.

Prior spatial information was also not incorporated within the nonlinear inversion algorithm, though it is used in many studies reported in the brain MRE literature (Hiscox, Johnson, McGarry, Schwarb, et al., 2020; Johnson et al., 2018; Schwarb et al., 2019). Soft prior regularization (SPR) is a method in which homogeneity in predefined spatial regions, typically obtained through an anatomical scan, is enforced through a penalty term in the nonlinear inversion strategy (McGarry et al., 2013). SPR increases sensitivity and decreases uncertainty in the assessment of smaller structures (Johnson et al., 2016); however, SPR performs better when there is low spatial variation across predefined brain regions. If substantial spatial variation is present, using SPR for that structure decreases repeatability relative to a fully distributed inversion (without SPR) (McGarry et al., 2013). In the present study, a large number of regions which have not previously been evaluated for SPR suitability (i.e., white matter tracts) were investigated and so SPR was not applied. In future work, regions demonstrated to be appropriate for SPR are likely to exhibit an increase in recovered contrast with surrounding tissue as well as improved repeatability.

The current study provides a new level of detail regarding brain mechanical properties in young adults, but there are a few issues to note. Finally, as we pooled data from multiple sites to provide more comprehensive population measures and build a more robust atlas, different scanner systems were used during data collection. To account for this, we included study as a fixed effect in our analyses. Variability between studies of up to 17% of the overall population mean was observed, depending on the region and measure; however, this variability is also likely due to the small populations included in each study (as small as six participants) and differences in their sex distributions. Future investigations may want to fully address the impact of scanner, frequency, and resolution effects, similar to previous studies that have investigated the impact of MR field strength in the brain (Hamhaber et al., 2010) or in how protocol variations can affect baseline measurements in liver MRE (Bohte et al., 2013; Reiter et al., 2020). These technical alternatives to data acquisition will be important aspects to consider in the event of the adoption of brain MRE as a clinical tool within neuroradiology.

## 5 CONCLUSIONS

In this study, standardized, in vivo atlases of the mechanical properties of the healthy human brain have been created. T1‐weighted structural images and high‐resolution MRE data from multiple studies were assembled and nonlinearly spatially normalized to a validated structural template. The resulting images contain new, emergent, anatomical detail that is consistent across participants. Through segmentation of different brain tissue types, we have performed a comprehensive analysis of the viscoelastic properties, and their variability, of various brain structures. Therefore, the data can be used as a reference to increase the diagnostic value of brain MRE for studies investigating neurological conditions and for informing computational models specific to traumatic brain injury and neurosurgery. The identification of sex differences in specific brain structures also suggest fundamentally different tissue microstructure exists between males and females. The high‐resolution templates within a standardized coordinate system are made openly available to the research community to foster collaboration across research groups and institutions and to support robust cross‐center comparisons. Future work will establish similar atlases in an older adult population that may assist future neuroimaging studies in assessing age‐related conditions such as Alzheimer's disease and other dementias.

## CONFLICT OF INTEREST

The authors have no actual or potential conflict of interest.

## DATA AVAILABILITY STATEMENT

The MRE templates are made openly available (github.com/mechneurolab/mre134) to foster collaboration across research institutions and to support robust cross‐center comparisons.

## Supporting information


**Table S1** Global MRE measures in native space.
**Table S2**. SGM MRE measures in native space.
**Table S3**. WMT MRE measures in native space.
**Table S4**. CGM MRE measures in native space.
