# Supplementary material for: Standard‐space atlas of the viscoelastic properties of the human brain
Source: Hum Brain Mapp. 2020 Sep 15;41(18):5282–300. doi: 10.1002/hbm.25192 (PMC7670638; doi:10.1002/hbm.25192)
Supplement: Supplementary file 1 — Table S1 Global MRE measures in native space. Table S2. SGM MRE measures in native space. Table S3. WMT MRE measures in native space. Table S4. CGM MRE measures in native space. [file HBM-41-5282-s001.docx]

**Standard-space atlas of the viscoelastic properties of the human brain**

Lucy V Hiscox, Matthew DJ McGarry, Hillary Schwarb, Elijah EW Van Houten, Ryan T Pohlig,

Neil Roberts, Graham R Huesmann, Agnieszka Z Burzynska, Bradley P Sutton, Charles H Hillman, Arthur F Kramer, Neal J Cohen, Aron K Barbey, Keith D Paulsen, and Curtis L Johnson

**Supp Table 1**. Global MRE measures in native space.

|  | Shear stiffness, μ (kPa) | | | Damping ratio, ξ | | |
| --- | --- | --- | --- | --- | --- | --- |
|  | Male | Female | Average | Male | Female | Average |
| Global | 2.84 ± 0.22 | 2.83 ± 0.22 | 2.83 ± 0.23 | 0.222 ± 0.022 | 0.225 ± 0.018 | 0.224 ± 0.020 |
| WM | 3.12 ± 0.23 | 3.09 ± 0.22 | 3.11 ± 0.24 | 0.224 ± 0.022 | 0.230 ± 0.018 | 0.227 ± 0.020 |
| SGM | 3.47 ± 0.39 | 3.47 ± 0.38 | 3.47 ± 0.40 | 0.200 ± 0.022 | 0.203 ± 0.026 | 0.201 ± 0.020 |
| CGM | 2.66 ± 0.22 | 2.65 ± 0.22 | 2.66 ± 0.23 | 0.231 ± 0.022 | 0.231 ± 0.026 | 0.231 ± 0.020 |

Mean + standard deviation (SD) are based on modified population marginal means supplied from the linear mixed model. Key: WM, white matter; SGM subcortical grey matter; CGM, cortical grey matter.

**Supp Table 2.** SGM MRE measures in native space.

|  | Shear stiffness, μ (kPa) | | | | Damping ratio, ξ | | |
| --- | --- | --- | --- | --- | --- | --- | --- |
|  | Male | Female | | Average | Male | Female | Average |
| AM | **3.51 ± 0.62** | | **3.26 ± 0.62** | 3.39 ± 0.64 | 0.173 ± 0.045 | 0.178 ± 0.044 | 0.176 ± 0.046 |
| CA | 3.25 ± 0.50 | | 3.12 ± 0.49 | 3.18 ± 0.52 | 0.228 ± 0.037 | 0.235 ± 0.044 | 0.231 ± 0.046 |
| HC | 2.73 ± 0.52 | | 2.85 ± 0.51 | 2.79 ± 0.54 | 0.173 ± 0.037 | 0.184 ± 0.035 | 0.178 ± 0.035 |
| PA | **4.06** ± **0.50** | | **3.87** ± **0.49** | 3.96 ± 0.52 | 0.188 ± 0.037 | 0.195 ± 0.035 | 0.191 ± 0.035 |
| PU | **4.00 ± 0.45** | | **3.84 ± 0.44** | 3.92 ± 0.46 | 0.191 ± 0.037 | 0.198 ± 0.035 | 0.195 ± 0.035 |
| TH | **3.51 ± 0.50** | | **3.31 ± 0.49** | 3.41 ± 0.52 | 0.198 ± 0.037 | 0.202 ± 0.035 | 0.200 ± 0.035 |

Mean + standard deviation (SD) are based on modified population marginal means supplied from the linear mixed model. Significant differences between males and females are indicated in bold and by *, p < 0.05. Key: AM, amygdala; CA, caudate; HC, hippocampus; PA, pallidum; PU, putamen; TH, thalamus.

**Supp Table 3:** WMT MRE measures in native space.

|  | Shear stiffness, μ (kPa) | | | Damping ratio, ξ | | |
| --- | --- | --- | --- | --- | --- | --- |
|  | Male | Female | Average | Male | Female | Average |
| ***Projection tracts*** | | | |  | |  |
| CST | **3.47** ± **0.40*** | **3.27** ± **0.40*** | 3.37 ± 0.41 | **0.205** ± **0.030*** | **0.218** ± **0.026*** | 0.211 ± 0.023 |
| ATR | 3.62 ± 0.38 | 3.51 ± 0.38 | 3.57 ± 0.39 | 0.220 ± 0.030 | 0.216 ± 0.026 | 0.218 ± 0.035 |
| PTR | 3.57 ± 0.34 | 3.57 ± 0.34 | 3.57 ± 0.35 | 0.216 ± 0.030 | 0.226 ± 0.026 | 0.221 ± 0.035 |
| CRa | 3.42 ± 0.32 | 3.35 ± 0.32 | 3.38 ± 0.33 | 0.247 ± 0.030 | 0.248 ± 0.035 | 0.247 ± 0.035 |
| ***Commissural tracts*** | | | | | | |
| CC | 3.04 ± 0.32 | 3.08 ± 0.32 | 3.06 ± 0.33 | 0.206 ± 0.030 | 0.204 ± 0.026 | 0.205 ± 0.023 |
| FMa | 3.16 ± 0.27 | 3.24 ± 0.27 | 3.20 ± 0.28 | 0.251 ± 0.037 | 0.245 ± 0.035 | 0.248 ± 0.035 |
| FMi | 3.27 ± 0.27 | 3.19 ± 0.26 | 3.23 ± 0.28 | 0.220 ± 0.030 | 0.214 ± 0.026 | 0.217 ± 0.035 |
| FX | 3.12 ± 0.57 | 2.94 ± 0.57 | 3.03 ± 0.58 | 0.226 ± 0.007 | 0.222 ± 0.006 | 0.224 ± 0.004 |
| ***Association tracts*** | | | | | | |
| UN | 3.45 ± 0.36 | 3.37 ± 0.35 | 3.41 ± 0.37 | 0.230 ± 0.045 | 0.244 ± 0.005 | 0.237 ± 0.046 |
| IFOF | 3.48 ± 0.29 | 3.47 ± 0.28 | 3.47 ± 0.30 | 0.222 ± 0.030 | 0.227 ± 0.026 | 0.225 ± 0.023 |
| ILF | 3.33 ± 0.31 | 3.30 ± 0.31 | 3.32 ± 0.32 | **0.222** ± **0.030*** | **0.238** ± **0.035*** | 0.230 ± 0.035 |
| SLF | 3.23 ± 0.33 | 3.16 ± 0.33 | 3.19 ± 0.34 | 0.237 ± 0.037 | 0.243 ± 0.044 | 0.240 ± 0.035 |

Mean + standard deviation (SD) based on modified population marginal means supplied from the linear mixed model. Significant differences between males and females are indicated in bold and by *, p < 0.05. Key: CST, corticospinal tract; ATR anterior thalamic radiation; PTR, posterior thalamic radiation; CRa, corona radiata; CC, corpus callosum; FMa, major forceps; FMi, minor forceps; FX, fornix; UN, uncinate; IFOF, inferior frontal-occipital fasciculus; ILF, inferior longitudinal fasciculus; SLF, superior longitudinal fasciculus.

**Supp Table 4:** CGM MRE measures in native space.

|  | Shear stiffness, μ (kPa) | | | Damping ratio, ξ | | |
| --- | --- | --- | --- | --- | --- | --- |
|  | Male | Female | Average | Male | Female | Average |
| ***Frontal lobe*** | | | | | | |
| SFC | 2.38 ± 0.24 | 2.33 ± 0.24 | 2.35 ± 0.25 | 0.187 ± 0.030 | 0.185 ± 0.026 | 0.186 ± 0.023 |
| RMF | 2.51 ± 0.23 | 2.45 ± 0.22 | 2.48 ± 0.23 | 0.280 ± 0.045 | 0.279 ± 0.044 | 0.279 ± 0.046 |
| PRE | **2.72 ± 0.29*** | **2.54 ± 0.29*** | 2.63 ± 0.30 | **0.235 ± 0.045*** | **0.255 ± 0.044*** | 0.245 ± 0.046 |
| ***Occipital lobe*** | | | | | | |
| LaO | 2.43 ± 0.19 | 2.47 ± 0.19 | 2.45 ± 0.20 | 0.246 ± 0.037 | 0.240 ± 0.035 | 0.243 ± 0.035 |
| LiO | 3.16 ± 0.25 | 3.15 ± 0.25 | 3.16 ± 0.26 | **0.102 ± 0.022*** | **0.109 ± 0.018*** | 0.106 ± 0.023 |
| CN | 2.47 ± 0.24 | 2.49 ± 0.24 | 2.48 ± 0.25 | **0.146 ± 0.030*** | **0.156 ± 0.026*** | 0.151 ± 0.023 |
| ***Parietal lobe*** | | | | | | |
| SPC | **2.23 ± 0.27*** | **2.08 ± 0.27*** | 2.16 ± 0.28 | 0.277 ± 0.037 | 0.276 ± 0.035 | 0.276 ± 0.035 |
| POST | **2.74 ± 0.30*** | **2.50 ± 0.30*** | 2.62 ± 0.31 | **0.255 ± 0.037*** | **0.272 ± 0.044*** | 0.263 ± 0.046 |
| PCN | 2.77 ± 0.30 | 2.73 ± 0.30 | 2.75 ± 0.31 | 0.129 ± 0.021 | 0.125 ± 0.018 | 0.129 ± 0.023 |
| ***Temporal lobe*** | | | | | | |
| STC | **2.89 ± 0.19*** | **2.78 ± 0.28*** | 2.83 ± 0.29 | **0.259 ± 0.045*** | **0.288 ± 0.044*** | 0.273 ± 0.046 |
| ITC | 2.68 ± 0.25 | 2.61 ± 0.24 | 2.64 ± 0.25 | 0.249 ± 0.052 | 0.266 ± 0.053 | 0.257 ± 0.046 |
| FSM | 2.77 ± 0.25 | 2.72 ± 0.25 | 2.75 ± 0.26 | **0.148 ± 0.030*** | **0.158 ± 0.026*** | 0.153 ± 0.035 |

Mean + standard deviation (SD) based on modified population marginal means supplied from the linear mixed model. Significant differences between males and females are indicated in bold and by *, *p* < 0.05. Key: superior frontal (SFC); rostral middle frontal (RMF); precentral (PRE); lateral occipital (LaO); lingual occipital (LiO); cuneus (CN); superior parietal (SPC); postcentral (POST); precuneus (PCN); superior temporal (STC); inferior temporal (ITC); fusiform (FSM).
